# Adjusting for Berkson error in exposure in ordinary and conditional logistic regression and in Poisson regression

**DOI:** 10.1186/s12874-023-02044-x

**Published:** 2023-10-10

**Authors:** Tamer Oraby, Santanu Chakraborty, Siva Sivaganesan, Laurel Kincl, Lesley Richardson, Mary McBride, Jack Siemiatycki, Elisabeth Cardis, Daniel Krewski

**Affiliations:** 1 School of Mathematical and Statistical Sciences, University of Texas Rio Grande Valley, Edinburg, TX USA; 2 Department of Mathematical Sciences, University of Cincinnati, Cincinnati, OH USA; 3 College of Health, Oregon State University, Corvallis, OR USA; 4 CRCHUM, Centre de Recherche Hospitalier de L’université de Montréal, Montreal, QC Canada; 5 BC Cancer Agency, Vancouver, BC Canada; 6 Barcelona Institute for Global Health (ISGlobal), Barcelona, Spain; 7 Pompeu Fabra University, Barcelona, Spain; 8 Spanish Consortium for Research and Public Health (CIBERESP), Instituto de Salud Carlos III, Madrid, Spain; 9 McLaughlin Centre for Population Health Risk Assessment, University of Ottawa, Ottawa, ON Canada; 10 Department of Epidemiology and Community Medicine, Faculty of Medicine, University of Ottawa, Ottawa, Canada; 11 Risk Sciences International, Ottawa, Canada

**Keywords:** Berkson error, Exposure surrogate, Electromagnetic fields, Brain cancer, Conditional logistic regression, Poisson regression

## Abstract

**Background:**

INTEROCC is a seven-country cohort study of occupational exposures and brain cancer risk, including occupational exposure to electromagnetic fields (EMF). In the absence of data on individual exposures, a Job Exposure Matrix (JEM) may be used to construct likely exposure scenarios in occupational settings. This tool was constructed using statistical summaries of exposure to EMF for various occupational categories for a comparable group of workers.

**Methods:**

In this study, we use the Canadian data from INTEROCC to determine the best EMF exposure surrogate/estimate from three appropriately chosen surrogates from the JEM, along with a fourth surrogate based on Berkson error adjustments obtained via numerical approximation of the likelihood function. In this article, we examine the case in which exposures are gamma-distributed for each occupation in the JEM, as an alternative to the log-normal exposure distribution considered in a previous study conducted by our research team. We also study using those surrogates and the Berkson error adjustment in Poisson regression and conditional logistic regression.

**Results:**

Simulations show that the introduced methods of Berkson error adjustment for non-stratified analyses provide accurate estimates of the risk of developing tumors in case of gamma exposure model. Alternatively, and under some technical assumptions, the arithmetic mean is the best surrogate when a gamma-distribution is used as an exposure model. Simulations also show that none of the present methods could provide an accurate estimate of the risk in case of stratified analyses.

**Conclusion:**

While our previous study found the geometric mean to be the best exposure surrogate, the present study suggests that the best surrogate is dependent on the exposure model; the arithmetic means in case of gamma-exposure model and the geometric means in case of log-normal exposure model. However, we could present a better method of Berkson error adjustment for each of the two exposure models. Our results provide useful guidance on the application of JEMs for occupational exposure assessments, with adjustment for Berkson error.

**Supplementary Information:**

The online version contains supplementary material available at 10.1186/s12874-023-02044-x.

## Background

In a retrospective cohort study, it is a challenge to find accurate measures of exposure to hazardous substances and radiation. Group-based exposure surrogates, such as those provided by job exposure matrices (JEM) in occupational epidemiological studies, are usually used to establish past exposures [1]. Another solution is to use a Berkson error model providing a model of the unobserved actual exposure via available exposure estimates, such as those derived from JEMs. While adjustment for Berkson error is an attractive concept, there remain open questions about the robustness of such approaches, as well as which exposure surrogates are best in different situations that may be encountered in practice.

Epidemiologists frequently make use of both prospective and retrospective cohort studies to identify risk factors for adverse health outcomes. In both cases, the goal is to identify risk factors that can discriminate between cases experiencing the adverse health effect of interest and controls who do not demonstrate this adverse effect. Mantel and Haenszel (1959) demonstrated the importance of stratification on covariates related to the outcome of interest and gave an estimator of the odds ratio formed by combining estimators from individual strata [2]. Truett, Cornfield and Cannel (1967) extended this pioneering work using a linear discriminant function to best discriminate between cases and controls for a given potential risk factor [3]. Day and Kerridge (1967) considered a method of discrimination based on maximum likelihood estimation that reduced the existing discriminant procedures to multivariate discriminant analysis [4]. The logistic discrimination function used by Day and Kerridge for dichotomous outcomes was generalized by Anderson (1972), in [5], to the polychotomous situation to accommodate three or more population groups. Prentice (1976) was the first to consider a binary logistic regression model for retrospective exposure probabilities that led to a direct estimate of the odds ratio [6]. This method was popularized in the well-known text on statistical methods for case–control studies by Breslow and Day (1980), in [7], which gave a detailed analysis of case–control studies and explained the advantages of conditional logistic regression over unconditional logistic regression [8]. Yanagawa (1979) provided in [9] an insightful discussion of the design of those types of studies, which up until this time had been retrospective in nature.

The notion of prospective studies in which exposed subjects would be followed to identify incident cases of the disease of interest was introduced by Prentice and Pyke (1979) in [10], extending previous work by Anderson (1972) in [5] and Breslow et al. (1978) in [11] for retrospective studies. In 1993, Wang and Carrol [12], generalized Prentice and Pyke’s results to robust logistic studies. Zhang (2006) subsequently extended this methodological work to a broader class of statistics using unbiased estimating equations [13].

Berkson error happens when using group exposure measurement in place of the actual individual measurements. This differs from classical measurement error, which arises due to inaccuracies in the measurement process. Berkson error does not lead to biased estimates in linear regression but can cause biased estimates of parameters in nonlinear models. Classical measurement error, on the other hand, can occur in various studies and can lead to biased estimates of relationships between variables. Both types of errors require careful identification in studies and the application of relevant statistical procedures to mitigate their effect on the results; otherwise, they may lead to misled conclusions. See [14] for more details about both types of errors.

In this article, we evaluate new approaches to adjusting JEM-based occupational exposure estimates for Berkson error in stratified and non-stratified analyses, using both logistic and Poisson regression. The main assumption is that variation in exposure about the true value follows a gamma distribution, a common choice in exposure modeling, e.g. [15, 16]. In [1], a non-stratified analysis using logistic regression was only considered under the assumption that the exposure is following a lognormal distribution. We determine the accuracy in the new methods and robustness of the exposure estimates to the change in assumptions using computer simulation. For the simulations to be relevant to real-world conditions, we use actual data from the INTEROCC study [17] to guide the simulation study. The theoretical and simulation components of this work are based on a maximum likelihood approach that facilitates Berkson error adjustment in extremely low frequency (ELF) electromagnetic field exposures. We show how the choice of the statistical model to describe exposure (lognormal versus gamma distributions) can affect the performance of the Berkson error adjustment and the exposure surrogates considered.

## Methods

### Canadian INTEROCC study

INTEROCC was a follow-up to the 13 country INTERPHONE [18] study of risk factors for brain cancer. While the primary goal of INTERPHONE was to investigate the association between brain cancer and use of mobile phones, socio-demographic, medical, occupational and other potential risk factors were also examined.

The INTEROCC study is a collaborative effort between 7 of these 13 countries (Australia, Canada, France, Germany, Israel, New Zealand, and the United Kingdom). One of the specific aims of INTEROCC was to investigate a possible association between occupational exposure to EMF and brain tumors (glioma and meningioma). This study used a JEM comprised of full-shift measurements of the TWA magnitudes of the ELF magnetic field B, in micro-tesla (μT). Each job was coded to the ISCO 1968 and 1988 occupational classification and each industry to the ISIC 1971 classification [19].

Out of 9,536 subjects in this cohort study, the Canadian component is comprised of 813 subjects of which 165 are brain cancer cases and the remaining 648 are controls. Each subject is classified by gender, education, age, and urban center. There are four education subclasses (primary-secondary, intermediate college, tertiary or do not know), four different age groups (< 40, 40—49, 50—50, and 60 + years of age), and three urban centers (Montreal, Ottawa, and Vancouver) within this database.

This study uses the Canadian INTEROCC database in a simulation study that simulates the brain cancer cases at different selected odd ratios based on simulating individual exposures using the job histories of the subjects. The odds ratios are then estimated using different exposure surrogates and the Berkson error adjustment described below. The study uses the JEM which consists of full-shift measurements of the TWA (time-weighted average) of the ELF magnetic field B, in micro-tesla (µT). The corresponding data were grouped by their ISCO (International Standard Classification of Occupation) codes [19]. The entries in the JEM were aggregated from different exposure studies to provide the arithmetic mean (AM) and standard deviation (SD), and the geometric mean (GM) and geometric standard deviation (GSD).

### Modeling exposures

Let the exposure ${\mathrm{X}}_{\mathrm{ij}}$ for the i^th^ subject in the j^th^ occupation be modeled using any probability density function $f\left(x\right)$. Here, we will use the gamma distribution.

The cumulative magnetic field (MF) exposures for subject $\mathrm{i}=1,\dots ,\mathrm{ N}$ is given as

1 $${\mathrm{CumMF}}_{\mathrm{i}}=\sum\nolimits_{j=1}^{{J}_{\mathrm{i}}}{\mathrm{t}}_{\mathrm{ij }}{\mathrm{X}}_{\mathrm{ij}},$$

where ${J}_{\mathrm{i}}$ is the number of jobs held by subject $\mathrm{i},$ ${\mathrm{t}}_{\mathrm{ij}}$ is the time (in years) spent by subject $\mathrm{i}$ in job $\mathrm{j}$ with annual exposure ${\mathrm{X}}_{\mathrm{ij}}$, and $\mathrm{N}$ is the number of subjects. We use a gamma probability distribution for ${\mathrm{X}}_{\mathrm{ij}}$ with shape parameter ${r}_{j}{=\mathrm{AM}}_{j}^{2}/{\mathrm{SD}}_{j}^{2}$ and rate ${\lambda }_{j}={\mathrm{AM}}_{j}/{\mathrm{SD}}_{j}^{2}$,which are determined using the JEM to determine${\mathrm{CumMF}}_{\mathrm{i}}$.

### Berkson error adjustment

The likelihood function in the Berkson error model is obtained by integrating across the exposure error distributions as

2 $$L\left(y\vert\beta_0,\beta_1,r,\lambda\right)=\int_0^\infty f\left(y\vert\beta_0,\beta_1,x\right)f\left(x\vert r,\lambda\right)dx.$$

(Here and elsewhere, boldface symbols are used to represent vectors and matrices.)

Also, let ${\varvec{y}}=\left({{\varvec{y}}}_{1},{{\varvec{y}}}_{2},\dots ,{{\varvec{y}}}_{{\varvec{N}}}\right)$ be the vector of responses of the $N$ subjects.

#### Ordinary logistic regression

In a non-stratified analysis, we would use ordinary logistic regression for which the following proposition is used for Berkson error adjustment.

##### Proposition 1

For ${\upbeta }_{0}\ge 0, {\upbeta }_{1}>0,$ and gamma exposure model, Eq. (2) can be expressed as

3 $$L\left({\varvec{y}}|{\beta }_{0},{\beta }_{1},{\varvec{r}},\boldsymbol{ }{\varvec{\lambda}}\right)=\prod_{i=1}^{N}{\left({\mathrm{p}}_{\mathrm{i}}\left({\varvec{r}},\boldsymbol{ }{\varvec{\lambda}}\right)\right)}^{{\mathrm{y}}_{\mathrm{i}}} {(1-{\mathrm{p}}_{\mathrm{i}}\left({\varvec{r}},\boldsymbol{ }{\varvec{\lambda}}\right))}^{{1-\mathrm{y}}_{\mathrm{i}}}$$

where

$${\mathrm{p}}_{\mathrm{i}}\left({\varvec{r}},\boldsymbol{ }{\varvec{\lambda}}\right)=\sum_{n=0}^{\infty }{(-1)}^{n}\mathrm{ exp}\left({-n\beta }_{0} \right)\frac{1}{\prod_{\mathrm{j}=1}^{{J}_{\mathrm{i}}}{\left(1+\frac{{n{\beta }_{1}{\mathrm{t}}_{\mathrm{ij }}\mathrm{SD}}_{j}^{2}}{{\mathrm{AM}}_{j}}\right)}^{{\mathrm{AM}}_{j}^{2}/{\mathrm{SD}}_{j}^{2}}}.$$

The proof of proposition 1 is given in Appendix I.

##### Remark

Modeling exposure with other probability distributions with moment generating function ${M}_{X}(t)$ (if it exists) will only affect the results in the right-hand side of Eq. (4). That is,

$$\underset{0}{\overset{\infty }{\int }}\mathrm{exp}\left(-n{\beta }_{1} {\mathrm{t}}_{\mathrm{ij }} {\mathrm{x}}_{\mathrm{ij }}\right) {\mathrm{f}}_{{\mathrm{X}}_{\mathrm{ij }}}\left({\mathrm{x}}_{\mathrm{ij }}\right)\mathrm{ d}{\mathrm{x}}_{\mathrm{ij }}= {\mathrm{M}}_{{\mathrm{X}}_{\mathrm{ij }}}\left(-n{\beta }_{1} {\mathrm{t}}_{\mathrm{ij}}\right)$$

and

$${\mathrm{p}}_{\mathrm{i}}\left({\varvec{r}},\boldsymbol{ }{\varvec{\lambda}}\right)=\sum_{n=0}^{\infty }{(-1)}^{n}\mathrm{ exp}\left({-n\beta }_{0} \right)\prod_{\mathrm{j}=1}^{{J}_{\mathrm{i}}}{\mathrm{M}}_{{\mathrm{X}}_{\mathrm{ij }}}\left(-n{\beta }_{1} {\mathrm{t}}_{\mathrm{ij }}\right) .$$

The gradient and Hessian of the log-likelihood of the adjusted ordinary logistic regression model is given in Appendix II.

##### Proposition 2

For the gamma exposure model with ${\upbeta }_{0}\ge 0\mathrm{ and }{\upbeta }_{1}>0,$ if $\mathrm{AM}/\mathrm{SD}$ is sufficiently large for all jobs, then the $\mathrm{AM}$ is the best surrogate (to approximate exposure) and

$$L\left({\varvec{y}}|{\beta }_{0},{\beta }_{1},{\varvec{r}},\boldsymbol{ }{\varvec{\lambda}}\right)= \prod_{i=1}^{N}{\left({\mathrm{p}}_{\mathrm{i}}\left(\mathbf{A}\mathbf{M}\right)\right)}^{{\mathrm{y}}_{\mathrm{i}}} {\left(1-{\mathrm{p}}_{\mathrm{i}}\left(\mathbf{A}\mathbf{M}\right)\right)}^{{1-\mathrm{y}}_{\mathrm{i}}}$$

where

$${\mathrm{p}}_{\mathrm{i}}\left(\mathbf{A}\mathbf{M}\right)= \frac{\mathrm{exp}\left({\beta }_{0}+{\beta }_{1} \sum_{j=1}^{{J}_{\mathrm{i}}}{\mathrm{t}}_{\mathrm{ij }}\;{\mathrm{AM}}_{j}\right)}{1+\mathrm{exp}\left({\beta }_{0}+{\beta }_{1} \sum_{j=1}^{{J}_{\mathrm{i}}}{\mathrm{t}}_{\mathrm{ij }}\;{\mathrm{AM}}_{j}\right)}.$$

#### Proof of proposition 2

Notice that when $\mathrm{AM}/\mathrm{SD}$ is very large, then

$$\prod\nolimits_{\mathrm{j}=1}^{{J}_{\mathrm{i}}}{\left(1+\frac{{n{\beta }_{1}{\mathrm{t}}_{\mathrm{ij }}\mathrm{SD}}_{j}^{2}}{{\mathrm{AM}}_{j}}\right)}^{{\mathrm{AM}}_{j}^{2}/{\mathrm{SD}}_{j}^{2}}\approx \prod\nolimits_{\mathrm{j}=1}^{{J}_{\mathrm{i}}}\mathrm{exp}\left(-n{\beta }_{1}{t}_{ij }{AM}_{j}\right)= \mathrm{exp}\left(-n{\beta }_{1}\sum\nolimits_{j=1}^{{J}_{\mathrm{i}}}{\mathrm{t}}_{\mathrm{ij }}{\mathrm{AM}}_{j}\right)$$

And

$$\underset{0}{\overset{\infty }{\iiint }}{\mathrm{p}}_{\mathrm{i}}\left(\mathrm{x}\right)\prod_{\mathrm{j}=1}^{{J}_{\mathrm{i}}}{\mathrm{f}}_{{\mathrm{X}}_{\mathrm{ij }}}\left({\mathrm{x}}_{\mathrm{ij }}\right)\mathrm{ d}{\mathrm{x}}_{\mathrm{ij }}\approx \sum_{n=0}^{\infty }{(-1)}^{n}\mathrm{ exp}\left({-n\beta }_{0} \right)\mathrm{exp}\left(-n{\beta }_{1}\sum_{j=1}^{{J}_{i}}{t}_{ij }{AM}_{j}\right)$$

$$=\frac{\mathrm{exp}\left({\beta }_{0}+{\beta }_{1} \sum_{j=1}^{{J}_{\mathrm{i}}}{\mathrm{t}}_{\mathrm{ij }} {\mathrm{AM}}_{j}\right)}{1+\mathrm{exp}\left({\beta }_{0}+{\beta }_{1} \sum_{j=1}^{{J}_{\mathrm{i}}}{\mathrm{t}}_{\mathrm{ij }} {\mathrm{AM}}_{j}\right)} .$$

This completes the proof. □

#### Poisson regression

For exposures ${\mathrm{X}}_{\mathrm{ij}}$ modeled using the gamma distribution given above and

$${Y}_{i}\sim Poisson\left(\Lambda \left({\beta }_{0},{\beta }_{1},{{\varvec{x}}}_{{\varvec{i}}}\right)\right)$$

where the rates $\Lambda \left({\beta }_{0},{\beta }_{1},{{\varvec{x}}}_{{\varvec{i}}}\right)$ are defined by

$$\Lambda \left({{\beta }_{0}, \beta }_{1},{{\varvec{x}}}_{{\varvec{i}}}\right)=E\left({Y}_{i}|{{\beta }_{0}, \beta }_{1},{{\varvec{x}}}_{{\varvec{i}}}\right)=\mathrm{exp}\left({{\beta }_{0}+\beta }_{1} \sum_{j=1}^{{J}_{\mathrm{i}}}{\mathrm{t}}_{\mathrm{ij }}{\mathrm{x}}_{\mathrm{ij}}\right)$$

we have the following proposition.

##### Proposition 3

For the gamma exposure model with ${\upbeta }_{0}\ge 0\mathrm{ and }{\upbeta }_{1}>0,$ Eq. (2) can be expressed as

4 $$L\left({\varvec{y}}|{\beta }_{0},{\beta }_{1},{\varvec{r}},\boldsymbol{ }{\varvec{\lambda}}\right)=\prod_{i=1}^{N}\frac{1}{{\mathrm{y}}_{\mathrm{i}}!}\mathrm{q}\left({\mathrm{y}}_{\mathrm{i}},{\varvec{r}},\boldsymbol{ }{\varvec{\lambda}}\right)$$

where

$$\mathrm{q}\left({\mathrm{y}}_{\mathrm{i}},{\varvec{r}},\boldsymbol{ }{\varvec{\lambda}}\right)=\sum_{n=0}^{\infty }\frac{{\left(-1\right)}^{n}}{\mathrm{n}!}\mathrm{exp}\left(({n{+\mathrm{y}}_{\mathrm{i}})\beta }_{0} \right)\prod_{\mathrm{j}=1}^{{J}_{\mathrm{i}}}\frac{1}{{\left(1-\frac{{({n{+\mathrm{y}}_{\mathrm{i}})\beta }_{1} {\mathrm{t}}_{\mathrm{ij }}\mathrm{SD}}_{j}^{2}}{{\mathrm{AM}}_{j}}\right)}^{\frac{{\mathrm{AM}}_{j}^{2}}{{\mathrm{SD}}_{j}^{2}}}}.$$

The proof of Proposition 3 is given in Appendix I.

##### Proposition 4

For the gamma exposure model with ${\upbeta }_{0}\ge 0\mathrm{ and }{\upbeta }_{1}>0,$ if $\mathrm{AM}/\mathrm{SD}$ is sufficiently large for all jobs, then the $\mathrm{AM}$ is the best surrogate (to approximate exposure) and

$$L\left({\varvec{y}}|{\beta }_{0},{\beta }_{1},{\varvec{r}},\boldsymbol{ }{\varvec{\lambda}}\right)= \prod_{i=1}^{N}\frac{1}{{\mathrm{y}}_{\mathrm{i}}!}{\left(\Lambda \left({\beta }_{0}, {\beta }_{1},\mathbf{A}\mathbf{M}\right)\right)}^{{\mathrm{y}}_{\mathrm{i}}} {e}^{-\Lambda \left({{\beta }_{0}, \beta }_{1},\mathbf{A}\mathbf{M}\right)}$$

where

$$\Lambda \left({\beta }_{0}, {\beta }_{1},\mathbf{A}\mathbf{M}\right)= \mathrm{exp}\left({{\beta }_{0}+\beta }_{1} \sum_{j=1}^{{J}_{\mathrm{i}}}{\mathrm{t}}_{\mathrm{ij }}{\mathrm{AM}}_{\mathrm{j}}\right).$$

##### Proof of proposition 4

The proof is similar to the proof of proposition 2.

#### Conditional logistic regression

If the $\mathrm{N}$ subjects are assigned to $\mathrm{S}$ strata according to covariates such as gender and age and there are ${N}_{k}$ control subjects for $k=1, 2,\dots , \mathrm{S}$, then the conditional likelihood under a logistic regression model is given by

5 $${L}_{C}\left({\beta }_{1}\right)= \prod_{k=1}^{S}\frac{\mathrm{exp}\left({\beta }_{1} \sum_{j=1}^{{J}_{0:\mathrm{k}}}{\mathrm{t}}_{0\mathrm{j}:\mathrm{k }}{\mathrm{x}}_{0\mathrm{j}:\mathrm{k}}\right)}{\mathrm{exp}\left({\beta }_{1} \sum_{j=1}^{{J}_{0:\mathrm{k}}}{\mathrm{t}}_{0\mathrm{j}:\mathrm{k }}{\mathrm{x}}_{0\mathrm{j}:\mathrm{k}}\right)+\sum_{i=1}^{{\mathrm{N}}_{k}}\mathrm{exp}\left({\beta }_{1} \sum_{j=1}^{{J}_{\mathrm{i}:\mathrm{k}}}{\mathrm{t}}_{\mathrm{ij}:\mathrm{k }}{\mathrm{x}}_{\mathrm{ij}:\mathrm{k}}\right)}$$

(Breslow and Day 1980) [8]. Here, $0:k$ and $0j:k$ refer to the case in stratum $k$ and the case in stratum $k$ with job index $j$; and $i:k$ and $ij:k$ refer to the control subject number $i$ in stratum $k$ and the control subject number $i$ in stratum $k$ with job index $j$.

In one situation, the conditional logistic likelihood is the same as the conditional Poisson likelihood with ${Y}_{i:k}\sim Poisson\left(\mathrm{exp}\left({\beta }_{1} \sum_{j=1}^{{J}_{\mathrm{i}:\mathrm{k}}}{\mathrm{t}}_{\mathrm{ij}:\mathrm{k }}{\mathrm{x}}_{\mathrm{ij}:\mathrm{k}}\right)\right)$ for ${i=0, 1, 2,\dots , N}_{k}$ (case and control subjects) for $k=1, 2,\dots , \mathrm{S}$, with the random variables ${Y}_{i:k}$ are independent for all $i$ and $k$. That situation happens when ${y}_{0:k}=1$ and ${y}_{1:k}=\dots ={y}_{{N}_{k}:k}=0$ for all $k$ ($k=1, 2,\dots , \mathrm{S}$).

Notice that for each $k$ ($k=1, 2,\dots , \mathrm{S}$)

$$\left[{Y}_{0:k}|{Y}_{0:k}+{Y}_{1:k}+\dots +{Y}_{{N}_{k}:k}=M\right]\sim \mathrm{Binomial}\left(M, \frac{\mathrm{exp}\left({\beta }_{1} \sum_{j=1}^{{J}_{0:\mathrm{k}}}{\mathrm{t}}_{0\mathrm{j}:\mathrm{k }}{\mathrm{x}}_{0\mathrm{j}:\mathrm{k}}\right)}{\mathrm{exp}\left({\beta }_{1} \sum_{j=1}^{{J}_{0:\mathrm{k}}}{\mathrm{t}}_{0\mathrm{j}:\mathrm{k }}{\mathrm{x}}_{0\mathrm{j}:\mathrm{k}}\right)+\sum_{i=1}^{{\mathrm{N}}_{k}}\mathrm{exp}\left({\beta }_{1} \sum_{j=1}^{{J}_{\mathrm{i}:\mathrm{k}}}{\mathrm{t}}_{\mathrm{ij}:\mathrm{k }}{\mathrm{x}}_{\mathrm{ij}:\mathrm{k}}\right)}\right).$$

Therefore,

$$P\left({Y}_{0:k}=1|{Y}_{0:k}+{Y}_{1:k}+\dots +{Y}_{{N}_{k}:k}=1\right)$$

$$=\frac{\mathrm{exp}\left({\beta }_{1} \sum_{j=1}^{{J}_{0:\mathrm{k}}}{\mathrm{t}}_{0\mathrm{j}:\mathrm{k }}{\mathrm{x}}_{0\mathrm{j}:\mathrm{k}}\right)}{\mathrm{exp}\left({\beta }_{1} \sum_{j=1}^{{J}_{0:\mathrm{k}}}{\mathrm{t}}_{0\mathrm{j}:\mathrm{k }}{\mathrm{x}}_{0\mathrm{j}:\mathrm{k}}\right)+\sum_{i=1}^{{\mathrm{N}}_{k}}\mathrm{exp}\left({\beta }_{1} \sum_{j=1}^{{J}_{\mathrm{i}:\mathrm{k}}}{\mathrm{t}}_{\mathrm{ij}:\mathrm{k }}{\mathrm{x}}_{\mathrm{ij}:\mathrm{k}}\right)}$$

which is the SoftMax function, leading to the aforementioned equivalence.

#### Berkson error adjustment for Poisson regression: (revisited)

Following (Prentice 1982) [6], for exposures ${\mathrm{X}}_{\mathrm{ij}}$ that can be modeled using the gamma distribution considered above and

$${Y}_{i}\sim Poisson\left(\mathrm{exp}\left({\beta }_{1} \sum_{j=1}^{{J}_{\mathrm{i}}}{\mathrm{t}}_{\mathrm{ij }}{\mathrm{x}}_{\mathrm{ij}}\right)\right)$$

we have

$$E\left({Y}_{i}|{\beta }_{1},{\varvec{x}}\right)=\mathrm{exp}\left({\beta }_{1} \sum_{j=1}^{{J}_{\mathrm{i}}}{\mathrm{t}}_{\mathrm{ij }}{\mathrm{x}}_{\mathrm{ij}}\right) .$$

Thus,

$$E\left({Y}_{i}|{\beta }_{1},{\varvec{r}},\boldsymbol{ }{\varvec{\lambda}}\right)=\underset{0}{\overset{\infty }{\iiint }}E\left({Y}_{i}|{\beta }_{1},{\varvec{x}}\right)\prod_{\mathrm{j}=1}^{{J}_{\mathrm{i}}}{\mathrm{f}}_{{\mathrm{X}}_{\mathrm{ij }}}\left({\mathrm{x}}_{\mathrm{ij }}\right)\mathrm{ d}{\mathrm{x}}_{\mathrm{ij}}$$

$$=\underset{0}{\overset{\infty }{\iiint }}\mathrm{exp}\left({\beta }_{1} \sum_{j=1}^{{J}_{\mathrm{i}}}{\mathrm{t}}_{\mathrm{ij }}{\mathrm{x}}_{\mathrm{ij}}\right)\prod_{\mathrm{j}=1}^{{J}_{\mathrm{i}}}{\mathrm{f}}_{{\mathrm{X}}_{\mathrm{ij }}}\left({\mathrm{x}}_{\mathrm{ij }}\right)\mathrm{ d}{\mathrm{x}}_{\mathrm{ij}}$$

$$=\prod_{\mathrm{j}=1}^{{J}_{\mathrm{i}}}\underset{0}{\overset{\infty }{\int }}\mathrm{exp}\left({\beta }_{1} {\mathrm{t}}_{\mathrm{ij }} {\mathrm{x}}_{\mathrm{ij }}\right) {\mathrm{f}}_{{\mathrm{X}}_{\mathrm{ij }}}\left({\mathrm{x}}_{\mathrm{ij }}\right)\mathrm{ d}{\mathrm{x}}_{\mathrm{ij }}=\frac{1}{\prod_{\mathrm{j}=1}^{{J}_{\mathrm{i}}}{\left(1-\frac{{{\beta }_{1}{\mathrm{t}}_{\mathrm{ij }}\mathrm{SD}}_{j}^{2}}{{\mathrm{AM}}_{j}}\right)}^{\frac{{\mathrm{AM}}_{j}^{2}}{{\mathrm{SD}}_{j}^{2}}}}$$

which exists when ${\upbeta }_{1} {\mathrm{t}}_{\mathrm{ij }}<{\mathrm{AM}}_{j}/{\mathrm{SD}}_{j}^{2}.$

##### Remark

Modeling exposure with other probability distributions with moment generating function ${M}_{X}(t)$ (if it exists) will only affect the results of the last equation. That is,

$$\underset{0}{\overset{\infty }{\int }}\mathrm{exp}\left({\beta }_{1} {\mathrm{t}}_{\mathrm{ij }} {\mathrm{x}}_{\mathrm{ij }}\right) {\mathrm{f}}_{{\mathrm{X}}_{\mathrm{ij }}}\left({\mathrm{x}}_{\mathrm{ij }}\right)\mathrm{ d}{\mathrm{x}}_{\mathrm{ij }}= {\mathrm{M}}_{{\mathrm{X}}_{\mathrm{ij }}}\left({\beta }_{1} {\mathrm{t}}_{\mathrm{ij }}\right)$$

and

$$E\left({Y}_{i}|{\beta }_{1}\right)=\prod_{\mathrm{j}=1}^{{J}_{\mathrm{i}}}{\mathrm{M}}_{{\mathrm{X}}_{\mathrm{ij }}}\left({\beta }_{1} {\mathrm{t}}_{\mathrm{ij }}\right).$$

In the following, we investigate adjusting for Berkson error in conditional logistic regression through the conditional Poisson likelihood and using

$${Y}_{i:k}\sim Poisson\left(\prod_{\mathrm{j}=1}^{{J}_{\mathrm{i}:\mathrm{k}}}{\left(1-\frac{{{\beta }_{1}{\mathrm{t}}_{\mathrm{ij}:\mathrm{k }}\mathrm{SD}}_{j}^{2}}{{\mathrm{AM}}_{j}}\right)}^{- \frac{{\mathrm{AM}}_{j}^{2}}{{\mathrm{SD}}_{j}^{2}}}\right)$$

for ${i=0, 1, 2,\dots , N}_{k}$ (case and control subjects) and $k=1, 2,\dots , \mathrm{S},$ that are independent for all $i$ and $k$ and when ${\upbeta }_{1} {\mathrm{t}}_{\mathrm{ij}:\mathrm{k }}<{\mathrm{AM}}_{j}/{\mathrm{SD}}_{j}^{2}$ for all $j$. Thus, the adjusted conditional logistic likelihood is

6 $${L}_{C,A}\left({\beta }_{1}\right)= \prod\nolimits_{k=1}^{S}\frac{\prod_{\mathrm{j}=1}^{{J}_{0:\mathrm{k}}}{\left(1-\frac{{{\beta }_{1}{\mathrm{t}}_{0\mathrm{j}:\mathrm{k }}\mathrm{SD}}_{j}^{2}}{{\mathrm{AM}}_{j}}\right)}^{- \frac{{\mathrm{AM}}_{j}^{2}}{{\mathrm{SD}}_{j}^{2}}}}{\prod_{\mathrm{j}=1}^{{J}_{0:\mathrm{k}}}{\left(1-\frac{{{\beta }_{1}{\mathrm{t}}_{0\mathrm{j}:\mathrm{k }}\mathrm{SD}}_{j}^{2}}{{\mathrm{AM}}_{j}}\right)}^{- \frac{{\mathrm{AM}}_{j}^{2}}{{\mathrm{SD}}_{j}^{2}}}+\sum_{i=1}^{{\mathrm{N}}_{k}}\prod_{\mathrm{j}=1}^{{J}_{\mathrm{i}:\mathrm{k}}}{\left(1-\frac{{{\beta }_{1}{\mathrm{t}}_{\mathrm{ij}:\mathrm{k }}\mathrm{SD}}_{j}^{2}}{{\mathrm{AM}}_{j}}\right)}^{- \frac{{\mathrm{AM}}_{j}^{2}}{{\mathrm{SD}}_{j}^{2}}}}.$$

The gradient and Hessian of the log likelihood of the conditional logistic function are given in Appendix III. In that case, the following lemma gives a condition for when the AM could be used as a surrogate.

##### Proposition 5

For a gamma exposure model, if $\mathrm{AM}/\mathrm{SD}$ is sufficiently large for all jobs, then the $\mathrm{AM}$ is the closest surrogate to the Berkson error adjustment.

##### Proof of proposition 5

When $\mathrm{AM}/\mathrm{SD}$ is large, we have

$$E\left({Y}_{i}|{\beta }_{1},{\varvec{r}},\boldsymbol{ }{\varvec{\lambda}}\right)=\frac{1}{\prod_{\mathrm{j}=1}^{{J}_{\mathrm{i}}}{\left(1-\frac{{{\beta }_{1}{\mathrm{t}}_{\mathrm{ij }}\mathrm{SD}}_{j}^{2}}{{\mathrm{AM}}_{j}}\right)}^{\frac{{\mathrm{AM}}_{j}^{2}}{{\mathrm{SD}}_{j}^{2}}}} \approx \frac{1}{\prod_{\mathrm{j}=1}^{{J}_{\mathrm{i}}}\mathrm{exp}\left(-{\beta }_{1}{\mathrm{t}}_{\mathrm{ij }}{\mathrm{AM}}_{j}\right)}=\mathrm{exp}\left({\beta }_{1}\sum_{j=1}^{{J}_{\mathrm{i}}}{\mathrm{t}}_{\mathrm{ij }}{\mathrm{AM}}_{j}\right).$$

Thus, using the conditional Poisson likelihood with

$${Y}_{i:k}\sim Poisson\left(\mathrm{exp}\left({\beta }_{1}\sum_{j=1}^{{J}_{\mathrm{i}:\mathrm{k}}}{\mathrm{t}}_{\mathrm{ij}:\mathrm{k }}{\mathrm{AM}}_{j}\right)\right)$$

ensures that the $\mathrm{AM}$ is the closest surrogate to the suggested Berkson adjustment in conditional logistic regression through conditional Poisson regression under the assumption of gamma exposure model.

### Simulation of non-stratified and stratified analyses

**Fig. 1:**
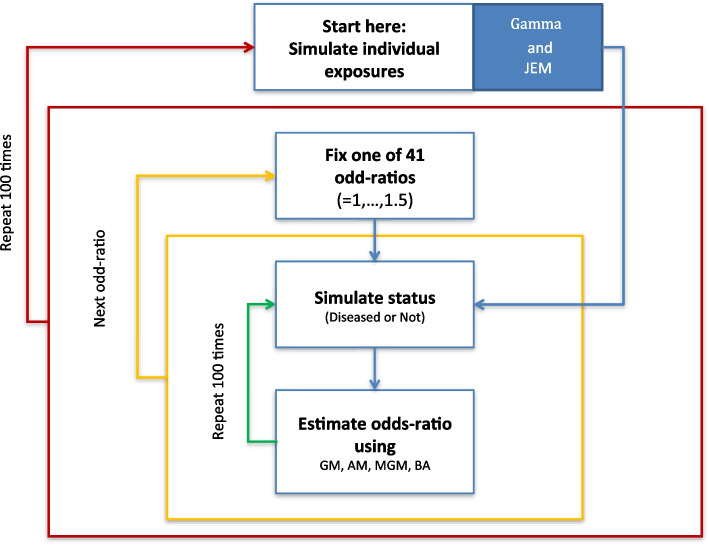
Schematic representation of the simulation study of odds ratio estimation using each of the following continuous exposure metrics: GM = geometric mean; AM = arithmetic mean; MGM = modified geometric mean; and BA = Berkson error adjustment using numerical integration with approximation degree $M=10$

Figure 1 gives an overview of the simulation study based on the 813 subjects in the Canadian component of the INTEROCC study with continuous exposure. In the non-stratified analysis, we use $M=10$ as the approximation degree for the Berkson error adjustment which showed quick convergence. The following calculations were performed in each simulation.

1. Occupational exposure for each subject is generated randomly according to ${X}_{ij}\sim Gamma\left({\left(\frac{{AM}_{j}}{{SD}_{j}}\right)}^{2},\frac{{\left({SD}_{j}\right)}^{2}}{{AM}_{j}}\right)$ for each job $j$ held by subject $i$. Here, for each $j$, ${AM}_{j}$ and ${SD}_{j}$ are provided by the JEM. The cumulative exposure, $Cum{MF}_{j}$ is then calculated for each $j$.
2. Using a pre-determined intercept ${\beta }_{0}=1$ and allowing a range of 0 to 0.4 for ${\beta }_{1}$ (with step-length = 0.01), the probability of developing a brain tumour is calculated as follows.

(a) For the non-stratified analysis, the probability ${p}_{i}$ that subject $i$ develops a brain tumor is calculated as:

$${p}_{i}=\frac{1}{1-\mathrm{exp}\left(-{\beta }_{0}-{\beta }_{1}Cum{MF}_{i}\right)}$$

for $$i=1,..., N.$$

(b) For the stratified analysis, the probability ${p}_{u:k}$ that subject $u$ in stratum $k$ develops a brain tumor is calculated as:

$${p}_{u:k}=\frac{\mathrm{exp}\left({\beta }_{1} \sum_{j=1}^{{J}_{0:\mathrm{k}}}{\mathrm{t}}_{\mathrm{uj}:\mathrm{k }}{\mathrm{x}}_{0\mathrm{j}:\mathrm{k}}\right)}{\mathrm{exp}\left({\beta }_{1} \sum_{j=1}^{{J}_{0:\mathrm{k}}}{\mathrm{t}}_{\mathrm{uj}:\mathrm{k }}{\mathrm{x}}_{0\mathrm{j}:\mathrm{k}}\right)+\sum_{i=1}^{{\mathrm{N}}_{k}}\mathrm{exp}\left({\beta }_{1} \sum_{j=1}^{{J}_{\mathrm{i}:\mathrm{k}}}{\mathrm{t}}_{\mathrm{ij}:\mathrm{k }}{\mathrm{x}}_{\mathrm{ij}:\mathrm{k}}\right)}$$

for $$u=1,..., {\mathrm{N}}_{k}$$ and $$k=1,..., S$$.

In both analyses, the range of slopes correspond to the range 1 to 1.5 for the odds ratios.

3. Simulation of cases and controls for the non-stratified and stratified designs was done as follows.
  - For the non-stratified analysis, we use a Bernoulli distribution with probability ${p}_{i}$ to randomly generate the case status of subject $i$.
  - For the stratified analysis, for each stratum $k$, we use a multinomial distribution with number of trials equal to one and probabilities (${p}_{u:k}$; $u=1,..., {\mathrm{N}}_{k}$) to generate one case and set the rest of the subjects in the stratum to be controls.
4. Using each of the statistics $AM$, $GM$, $MGM$ as a proxy for the actual exposure, the slope of the exposure–response curve (the logarithm of the odds ratio) is then estimated. We then apply a Berkson error adjustment based on Proposition 1 for the non-stratified analysis and Proposition 5 for the stratified analysis to estimate brain tumour risk.
5. We repeat steps 3 and 4 for 100 times and calculate the median estimate for the 100 estimates of the pre-determined slope. (The median estimate is chosen as the measure of central tendency as the distribution is right skewed for each of the pre-determined slope values.)
6. Next, we repeat steps 1 through 5 for 100 times and calculate the mean, the 2.5% percentile, and the 97.5% percentile from the distribution of slope estimates.

For comparing the five different approaches to risk estimation based on different exposure surrogates with one another, the bias defined by the average (over all simulation runs) risk estimate minus the pre-determined target parameter was calculated. The root mean-square error, given by the square root of the sum of the variance estimates and the square of the bias were also calculated. The variance here is the total variance calculated as the sum of the following two terms: one is the mean or average of the conditional variances, and the other is the variance of the conditional means with the simulated inputs being the condition in both these terms.

We did not perform a simulation study for Poisson regression as we expect the results to be essentially the same as those for ordinary logistic regression.

### Results of the simulation study

**Fig. 2:**
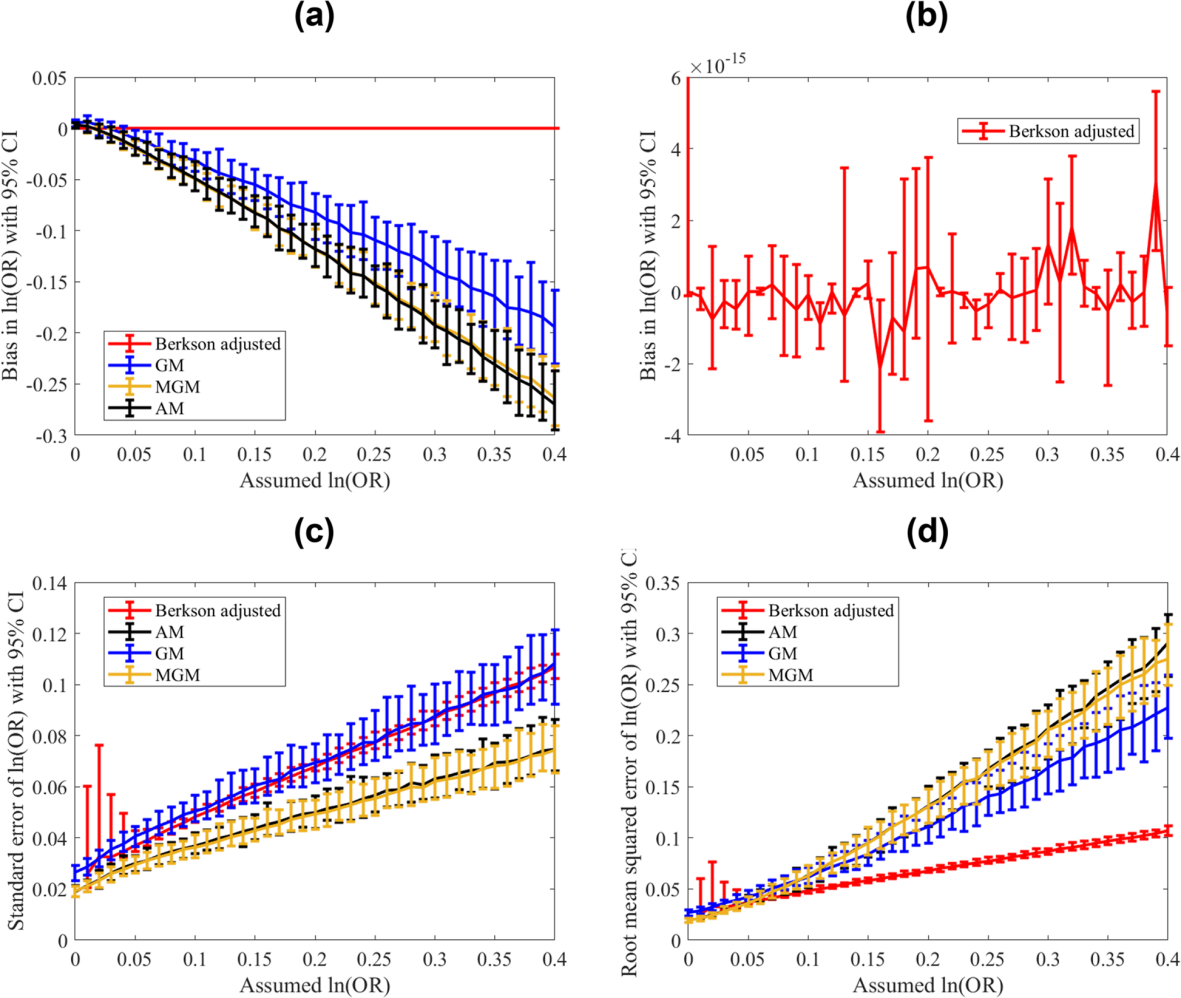
Bias in the estimates of (**a**) the log odds ratios using the four-exposure metrics: GM = geometric mean, AM = arithmetic mean, MGM = log-normal mean, and Berkson error adjustment using numerical integration. **b** The same outputs are shown only for the best approach: Berkson error adjustment using numerical integration for (**c**) the standard error (**d**) the root mean square error

Using ordinary logistic regression and the approach described in Proposition 1, we observe that the Berkson adjusted surrogate shows the minimum bias (as depicted in Fig. 2a). It has, moreover, a negligible bias, see Fig. 2b. The AM and Berkson adjusted surrogate perform similarly with respect to standard error, with the AM being the slightly better surrogate (see Proposition 2). Yet, the root mean squared error of the Berkson error adjustment compensates for that slight better precision, see Fig. 2d.

**Fig. 3:**
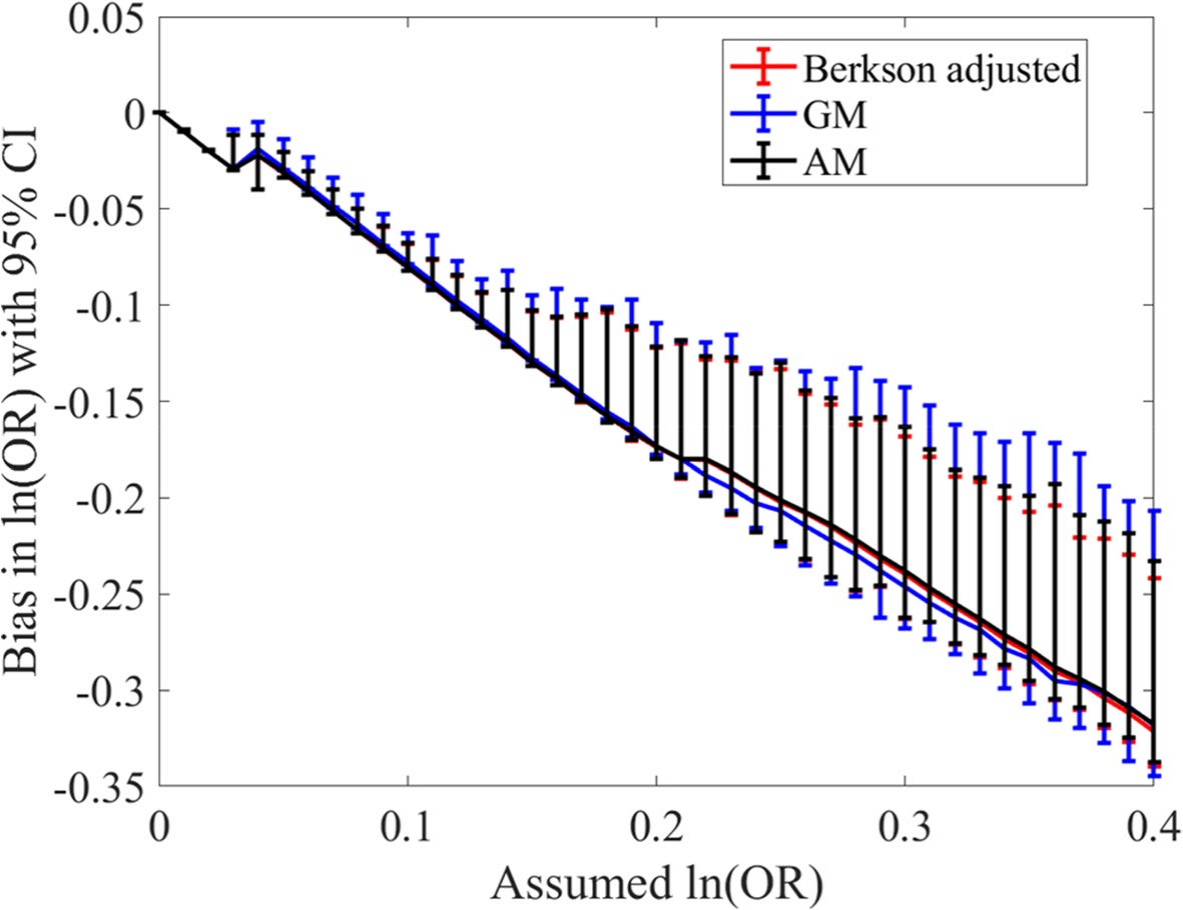
Bias in the estimates of the log odds ratios using the three-exposure metrics: GM = geometric mean, AM = arithmetic mean, and Berkson error adjustment using numerical integration

Proposition 5 ensures that the $\mathrm{AM}$ to be the closest surrogate to using the suggested Berkson adjustment in conditional logistic regression under the assumption of gamma exposure model. That idea is observed in the simulation results by both showing very close degrees of bias in estimates of the logarithms of the odd-ratios. Yet, neither one of them shows any improvement in estimating the logarithms of the odd-ratios (see Fig. 3). Moreover, the GM gives very close estimates to theirs. That would indicate that using AM or GM as surrogates in stratified analyses are not leading to unbiased estimates.

## Discussion

Many retrospective cohort studies face the challenge of ascertaining exposures prior to diagnosis of the disease of interest. In the absence of direct measurement of occupational exposures, exposure models are often assumed by researchers to compensate for data unavailability. A Berkson error model combined with job exposure matrices represents one such exposure model, with a Berkson error adjustment used to correct for the ensuing bias and increase in variability.

In this paper, we used numerical integration in Berkson error models for both ordinary and conditional logistic regression to adjust for Berkson error in occupational exposure estimates derived from JEMs. We also considered Poisson regression as another statistical model. We also carried out simulation studies were guided by data from the Canadian component of the INTEROCC study of the association between EMF and brain cancer. In all cases considered, we assumed that the amount of ELF exposure follows a gamma distribution. In the ordinary logistic analysis approach the Berkson error adjustment was successful in generating estimates with the lowest bias and mean-squared error (MSE).

In previous work, Oraby et al. (2018) [1] considered the distribution of the exposure during each job to be lognormal instead of gamma. For the bias comparisons with ordinary logistic regression in the lognormal scenario, both GM and Berkson adjusted surrogates performed equally well, whereas in the current gamma scenario, the Berkson adjusted surrogate outperforms all other surrogates. For the root mean squared error comparisons, the GM and the Berkson adjusted surrogate are jointly best in the lognormal case, but the Berkson adjusted surrogate uniquely outperforms other exposure surrogates, except for some small values of the log likelihood function in which the AM is better. With regards to standard error comparisons, these two are once again the best in the lognormal case, with the GM slightly outperforming the Berkson adjusted surrogate; the AM and the Berkson adjusted surrogate are the two best surrogates in the gamma case with the AM slightly outperforming (only for smaller values) the Berkson adjusted surrogate. The case of GM for lognormal distribution and AM for gamma distribution might be due to that they are sufficient statistics for some of their parameters. Epidemiologists must consult the literature of the exposure type and decide upon the appropriate exposure model or use an external exposure study. If there is not enough information about the exposure, then both AM and GM must be used since each one of them can give a different conclusion.

Some epidemiological stratified analyses use the arithmetic mean and the geometric mean as surrogates in retrospective cohort studies. In those studies, researchers use conditional logistic regression as described in this paper. We have shown that in that case Berkson adjustment as well as using the arithmetic mean and the geometric mean as surrogates do not provide accurate estimates of the logarithms of the odds-ratios. That must shed some light on the challenge in finding models of exposures for stratified analyses in those cohort studies.

We have shown that using Berkson error adjustment and surrogates when the statistical analyses are done using conditional logistic regression lead to inaccurate estimates. Hence, there remains a need to find accurate and precise exposure surrogates that can be reliably used in conditional logistic regression. Berkson error adjustment for other regression models, such as the Cox proportional hazard model, and other models of exposure, such as power law models, are also important open research topics.

## Conclusions

The results presented in this paper show that in case of gamma exposure models, using methods of Berkson error adjustment are far better than using surrogates. That conclusion along with our earlier results about the case of log-normal exposure model [1] support the conclusion that the presented Berkson error adjustment methods are more accurate, and show be directly used. The conclusions in this paper raise doubts about the results of epidemiological studies based on stratified and non-stratified analyses that use surrogates from job exposure matrices without validating the assumptions discussed here and in our earlier paper. They also provide a solution to them in case of non-stratified analyses, whereas the case of stratified analyses remains an open problem.

### Supplementary Information

**Additional file 1: Appendix I.** Proofs of Propositions 1 and 3. **Appendix II.** Gradient and Hessian of the log-likelihood of the ordinary logistic regression. **Appendix III.** Gradient and Hessian of the log-likelihood of the conditional logistic regression.

## Data Availability

The datasets generated and/or analyzed during the current study are not publicly available due to the requirements of the pertinent Institutional Review Boards but are available from the corresponding author on reasonable request.

## Abbreviations

AM: Arithmetic mean
BA: Berkson error adjustment
ELF: Extremely low frequency
EMF: Electromagnetic field
GM: Geometric mean
GSD: Geometric standard deviation
INTEROCC: International Occupational Study
JEM: Job exposure matrix
MGM: Modified geometric mean
MSE: Mean square error
SD: Standard deviation
TWA: Time-weighted average
