# Supplementary material for: Adjusting for Berkson error in exposure in ordinary and conditional logistic regression and in Poisson regression
Source: BMC Med Res Methodol. 2023 Oct 10;23:225. doi: 10.1186/s12874-023-02044-x (PMC10566152; doi:10.1186/s12874-023-02044-x)
Supplement: Supplementary file 1 — Additional file 1: Appendix I. Proofs of Propositions 1 and 3. Appendix II. Gradient and Hessian of the log-likelihood of the ordinary logistic regression. Appendix III. Gradient and Hessian of the log-likelihood of the conditional logistic regression. [file 12874_2023_2044_MOESM1_ESM.docx]

**Appendix I: Proofs of Propositions 1 and 3.**

**Proof of Proposition 1:** The proof is based on Taylor expansion and some straightforward algebraic manipulations.

Let ${p_{i}(X):=p}_{i}\left( X_{\mathrm{ij}};j=1,\ldots,J_{i} \right)$ be the probability of being diagnosed with the disease $Pr\left( y_{i}=1 \right)$. That is, for $i$ $(i=1,\ldots, N)$

| $p_{i}(X)=\frac{\exp\left( \beta_{0}+\beta_{1} \sum_{j=1}^{J_{i}} t_{\mathrm{ij}}X_{\mathrm{ij}} \right)}{1+exp\left( \beta_{0}+\beta_{1} \sum_{j=1}^{J_{i}} t_{\mathrm{ij}}X_{\mathrm{ij}} \right)} .$ |  |
| --- | --- |

First, it can be seen that

| $L\left( \boldsymbol{y} \vert\beta_{0},\beta_{1},\boldsymbol{r, \lambda} \right)=\iiint_{0}^{\infty} \prod_{i=1}^{N} {p_{i}\left( x \right)}^{y_{i}}\left( 1-p_{i}\left( x \right) \right)^{{1-y}_{i}}\prod_{j=1}^{J_{i}} f_{X_{\mathrm{ij}}}\left( x_{\mathrm{ij}} \right) dx_{\mathrm{ij}}=\prod_{i=1}^{N} \iiint_{0}^{\infty} {p_{i}\left( x \right)}^{y_{i}}\left( 1-p_{i}\left( x \right) \right)^{{1-y}_{i}}\prod_{j=1}^{J_{i}} f_{X_{\mathrm{ij}}}\left( x_{\mathrm{ij}} \right) dx_{\mathrm{ij}}=\prod_{i=1}^{N} \left( \iiint_{0}^{\infty} p_{i}\left( x \right)\prod_{j=1}^{J_{i}} f_{X_{\mathrm{ij}}}\left( x_{\mathrm{ij}} \right) dx_{\mathrm{ij}} \right)^{y_{i}}* \left( 1-\iiint_{0}^{\infty} p_{i}\left( x \right)\prod_{j=1}^{J_{i}} f_{X_{\mathrm{ij}}}\left( x_{\mathrm{ij}} \right) dx_{\mathrm{ij}} \right)^{{1-y}_{i}} ,$ |  |
| --- | --- |

where the first equality is due to the independence of exposures and the last equality is a consequence to the fact that $y_{i}=0$ or$1$. Note that

$$\iiint_{0}^{\infty} p_{i}\left( x \right)\prod_{j=1}^{J_{i}} f_{X_{\mathrm{ij}}}\left( x_{\mathrm{ij}} \right) dx_{\mathrm{ij}}=\iiint_{0}^{\infty} \frac{\exp\left( \beta_{0}+\beta_{1} \sum_{j=1}^{J_{i}} t_{\mathrm{ij}}x_{\mathrm{ij}} \right)}{1+exp\left( \beta_{0}+\beta_{1} \sum_{j=1}^{J_{i}} t_{\mathrm{ij}} x_{\mathrm{ij}} \right)}\prod_{j=1}^{J_{i}} f_{X_{\mathrm{ij}}}\left( x_{\mathrm{ij}} \right) dx_{\mathrm{ij}}$$

where $X_{\mathrm{ij}}$is $\text{Gamma}\left( r_{j},\lambda_{j} \right)$, with $r_{j}{=AM}_{j}^{2}/\mathrm{SD}_{j}^{2}$ and $\lambda_{j}=\mathrm{AM}_{j}/\mathrm{SD}_{j}^{2}$. Since $\beta_{0}\geq0, \beta_{1}>0, \text{and} t_{\mathrm{ij}},X_{\mathrm{ij}}>0$ for all$j$, then

$$\frac{\exp\left( \beta_{0}+\beta_{1} \sum_{j=1}^{J_{i}} t_{\mathrm{ij}} X_{\mathrm{ij}} \right)}{1+exp\left( \beta_{0}+\beta_{1} \sum_{j=1}^{J_{i}} t_{\mathrm{ij}} X_{\mathrm{ij}} \right)}=\left( 1+exp\left( {-\beta}_{0}-\beta_{1} \sum_{j=1}^{J_{i}} t_{\mathrm{ij}} X_{\mathrm{ij}} \right) \right)^{-1}=\sum_{n=0}^{\infty} {(-1)}^{n} exp\left( {-n\beta}_{0}-n\beta_{1} \sum_{j=1}^{J_{i}} t_{\mathrm{ij}} X_{\mathrm{ij}} \right).$$

Thus,

$$\iiint_{0}^{\infty} p_{i}\left( x \right)\prod_{j=1}^{J_{i}} f_{X_{\mathrm{ij}}}\left( x_{\mathrm{ij}} \right) dx_{\mathrm{ij}}=\iiint_{0}^{\infty} \sum_{n=0}^{\infty} {(-1)}^{n}\exp\left( {-n\beta}_{0}-n\beta_{1} \sum_{j=1}^{J_{i}} t_{\mathrm{ij}}x_{\mathrm{ij}} \right)\prod_{j=1}^{J_{i}} f_{X_{\mathrm{ij}}}\left( x_{\mathrm{ij}} \right) dx_{\mathrm{ij}}=\sum_{n=0}^{\infty} {(-1)}^{n}\iiint_{0}^{\infty} \exp\left( {-n\beta}_{0}-n\beta_{1} \sum_{j=1}^{J_{i}} t_{\mathrm{ij}} x_{\mathrm{ij}} \right) \prod_{j=1}^{J_{i}} f_{X_{\mathrm{ij}}}\left( x_{\mathrm{ij}} \right) dx_{\mathrm{ij}}=\sum_{n=0}^{\infty} {(-1)}^{n} exp\left( {-n\beta}_{0} \right)\prod_{j=1}^{J_{i}} \int_{0}^{\infty} exp(-n\beta_{1} t_{\mathrm{ij}} x_{\mathrm{ij}}) f_{X_{\mathrm{ij}}}\left( x_{\mathrm{ij}} \right) dx_{ij .}$$

The second equality is due to Fubini’s theorem, where $0<exp(-x)\leq1$ for $x\geq0$. Note that the integral in the last step is the moment generating function of a gamma random variable, which exists since$-n\beta_{1} t_{\mathrm{ij}}<\lambda_{j}=\mathrm{AM}_{j}/\mathrm{SD}_{j}^{2}$, and is given by

| $\int_{0}^{\infty} \exp\left( -n\beta_{1} t_{\mathrm{ij}} x_{\mathrm{ij}} \right) f_{X_{\mathrm{ij}}}\left( x_{\mathrm{ij}} \right) dx_{\mathrm{ij}}= \frac{1}{\left( 1+\frac{{n\beta_{1}t_{\mathrm{ij}}\mathrm{SD}}_{j}^{2}}{\mathrm{AM}_{j}} \right)^{\frac{\mathrm{AM}_{j}^{2}}{\mathrm{SD}_{j}^{2}}}}.$ | (4) |
| --- | --- |

Therefore,

$$\iiint_{0}^{\infty} p_{i}\left( x \right)\prod_{j=1}^{J_{i}} f_{X_{\mathrm{ij}}}\left( x_{\mathrm{ij}} \right) dx_{\mathrm{ij}}=\sum_{n=0}^{\infty} \left( -1 \right)^{n}\exp\left( {-n\beta}_{0} \right)\frac{1}{\prod_{j=1}^{J_{i}} \left( 1+\frac{{n\beta_{1}t_{\mathrm{ij}}\mathrm{SD}}_{j}^{2}}{\mathrm{AM}_{j}} \right)^{\frac{\mathrm{AM}_{j}^{2}}{\mathrm{SD}_{j}^{2}}}}.$$

This completes the proof. □

**Proof of Proposition 3:** Again, the proof is based on Taylor’s expansion and some elementary manipulations. The likelihood function can be written as

| $L\left( \boldsymbol{y} \vert\beta_{0},\beta_{1},\boldsymbol{r, \lambda} \right)=\iiint_{0}^{\infty} \prod_{i=1}^{N} \frac{1}{y_{i}!}\left( \Lambda\left( \beta_{0}, \beta_{1}\boldsymbol{,}\boldsymbol{x}_{\boldsymbol{i}} \right) \right)^{y_{i}} e^{-\Lambda\left( {\beta_{0}, \beta}_{1}\boldsymbol{,}\boldsymbol{x}_{\boldsymbol{i}} \right)}\prod_{j=1}^{J_{i}} f_{X_{\mathrm{ij}}}\left( x_{\mathrm{ij}} \right) dx_{\mathrm{ij}}=\iiint_{0}^{\infty} \prod_{i=1}^{N} \frac{1}{y_{i}!}\sum_{n=0}^{\infty} \frac{\left( -1 \right)^{n}}{n!}\mathrm{ex}p \left( ({n{+y}_{i})\beta}_{0} \right)\exp\left( ({n{+y}_{i})\beta}_{1} \sum_{j=1}^{J_{i}} t_{\mathrm{ij}}x_{\mathrm{ij}} \right)\prod_{j=1}^{J_{i}} f_{X_{\mathrm{ij}}}\left( x_{\mathrm{ij}} \right) dx_{\mathrm{ij}}=\prod_{i=1}^{N} \frac{1}{y_{i}!}\sum_{n=0}^{\infty} \frac{\left( -1 \right)^{n}}{n!}\mathrm{ex}p \left( ({n{+y}_{i})\beta}_{0} \right)\iiint_{0}^{\infty} \prod_{j=1}^{J_{i}} \exp\left( ({n{+y}_{i})\beta}_{1} t_{\mathrm{ij}}x_{\mathrm{ij}} \right)f_{X_{\mathrm{ij}}}\left( x_{\mathrm{ij}} \right) dx_{\mathrm{ij}}$ |  |
| --- | --- |

where the equalities are due to the independence of subjects and exposures and where $X_{\mathrm{ij}}$is $\text{Gamma}\left( r_{j},\lambda_{j} \right)$, with $r_{j}{=AM}_{j}^{2}/\mathrm{SD}_{j}^{2}$ and $\lambda_{j}=\mathrm{AM}_{j}/\mathrm{SD}_{j}^{2}$. Since $\beta_{0}\geq0, \beta_{1}>0, \text{and} t_{\mathrm{ij}},X_{\mathrm{ij}}>0$ for all$j$, then

$$\iiint_{0}^{\infty} \prod_{j=1}^{J_{i}} \exp\left( ({n{+y}_{i})\beta}_{1} t_{\mathrm{ij}}x_{\mathrm{ij}} \right)f_{X_{\mathrm{ij}}}\left( x_{\mathrm{ij}} \right) dx_{\mathrm{ij}} =\prod_{j=1}^{J_{i}} \int_{0}^{\infty} \exp\left( ({n{+y}_{i})\beta}_{1} t_{\mathrm{ij}}x_{\mathrm{ij}} \right) f_{X_{\mathrm{ij}}}\left( x_{\mathrm{ij}} \right)dx_{\mathrm{ij}}$$

$$=\prod_{j=1}^{J_{i}} \int_{0}^{\infty} \exp\left( ({n{+y}_{i})\beta}_{1} t_{\mathrm{ij}}x_{\mathrm{ij}} \right) f_{X_{\mathrm{ij}}}\left( x_{\mathrm{ij}} \right)dx_{\mathrm{ij}} .$$

Note that the integral in the last step is the moment generating function of a gamma random variable which exists when$({n{+y}_{i})\beta}_{1} t_{\mathrm{ij}}<\lambda_{j}=\mathrm{AM}_{j}/\mathrm{SD}_{j}^{2}$, and is given by

| $\int_{0}^{\infty} \exp\left( ({n{+y}_{i})\beta}_{1}t_{\mathrm{ij}} x_{\mathrm{ij}} \right) f_{X_{\mathrm{ij}}}\left( x_{\mathrm{ij}} \right) dx_{\mathrm{ij}}= \frac{1}{\left( 1-\frac{{({n{+y}_{i})\beta}_{1} t_{\mathrm{ij}}\mathrm{SD}}_{j}^{2}}{\mathrm{AM}_{j}} \right)^{\frac{\mathrm{AM}_{j}^{2}}{\mathrm{SD}_{j}^{2}}}}$. | (4) |
| --- | --- |

Therefore,

$$L\left( \boldsymbol{y} | \beta_{0},\beta_{1},\boldsymbol{r, \lambda} \right)=\prod_{i=1}^{N} \frac{1}{y_{i}!}\sum_{n=0}^{\infty} \frac{\left( -1 \right)^{n}}{n!}\mathrm{ex}p \left( ({n{+y}_{i})\beta}_{0} \right)\prod_{j=1}^{J_{i}} \frac{1}{\left( 1-\frac{{({n{+y}_{i})\beta}_{1} t_{\mathrm{ij}}\mathrm{SD}}_{j}^{2}}{\mathrm{AM}_{j}} \right)^{\frac{\mathrm{AM}_{j}^{2}}{\mathrm{SD}_{j}^{2}}}}.$$

This completes the proof. □

**Appendix II: Gradient and Hessian of the log-likelihood of the ordinary logistic regression**

Notice that $f^{'}\left( x \right)=f\left( x \right)\left( \log f(x) \right)'$ and so

$$\frac{d}{d\beta} \prod_{j=1}^{J} g_{j}\left( \beta\right)=\prod_{j=1}^{J} g_{j}\left( \beta\right)*\sum_{j=1}^{J} \frac{g_{j}'\left( \beta\right)}{g_{j}\left( \beta\right)}$$

The likelihood is given by

| $L\left( {\beta_{0},\beta}_{1} \right):=L\left( y \vert\beta_{0},\beta_{1},\boldsymbol{r, \lambda} \right)=\prod_{i=1}^{N} \left( p_{i}\left( \boldsymbol{r, \lambda} \right) \right)^{y_{i}} \left( 1-p_{i}\left( \boldsymbol{r, \lambda} \right) \right)^{{1-y}_{i}}$  $=\prod_{i=1}^{N} \left( 1-y_{i}+\left( -1 \right)^{1-y_{i}} *p_{i}\left( \boldsymbol{r, \lambda} \right) \right)$ |  |
| --- | --- |

where

$$p_{i}\left( \boldsymbol{r, \lambda} \right)=\sum_{n=0}^{\infty} {(-1)}^{n} exp\left( {-n\beta}_{0} \right)\frac{1}{\prod_{j=1}^{J_{i}} \left( 1+\frac{{n\beta_{1}t_{\mathrm{ij}}\mathrm{SD}}_{j}^{2}}{\mathrm{AM}_{j}} \right)^{\mathrm{AM}_{j}^{2}/\mathrm{SD}_{j}^{2}}}$$

Denote

$$\varphi_{ij}\left( n,\beta_{1} \right)=1+\frac{{n\beta_{1}t_{\mathrm{ij}}\mathrm{SD}}_{j}^{2}}{\mathrm{AM}_{j}},$$

$\Phi_{i}\left( n,\beta_{1} \right)=\frac{1}{\prod_{j=1}^{J_{i}} \left( 1+\frac{{n\beta_{1}t_{\mathrm{ij}}\mathrm{SD}}_{j}^{2}}{\mathrm{AM}_{j}} \right)^{\mathrm{AM}_{j}^{2}/\mathrm{SD}_{j}^{2}}}$

so that

$$p_{i}\left( \boldsymbol{r, \lambda} \right)=\sum_{n=0}^{\infty} {(-1)}^{n} exp\left( {-n\beta}_{0} \right)\Phi_{i}\left( n,\beta_{1} \right)\ldots\ldots(1)$$

Note that,

$$\frac{\partial}{\partial\beta_{0}}p_{i}\left( \boldsymbol{r, \lambda} \right)= \sum_{n=0}^{\infty} n*{(-1)}^{n+1} exp\left( {-n\beta}_{0} \right)\Phi_{i}\left( {n,\beta}_{1} \right)\ldots\ldots(2)$$

Now

$$\frac{\partial}{\partial\beta_{1}}\left[ \varphi_{ij}\left( \beta_{1} \right) \right]=\frac{{nt_{\mathrm{ij}}\mathrm{SD}}_{j}^{2}}{\mathrm{AM}_{j}}$$

$$\Rightarrow\frac{\partial}{\partial\beta_{1}}\left[ \Phi_{i}\left( \beta_{1} \right) \right]=\Phi_{i}\left( \beta_{1} \right)\frac{\partial}{\partial\beta_{1}}\left[ \log\left( \Phi_{i}\left( \beta_{1} \right) \right) \right]=\Phi_{i}\left( \beta_{1} \right)\left[ -\sum_{j=1}^{J_{i}} \frac{{{AM}_{j}}^{2}}{{{SD}_{j}}^{2}}\frac{\partial}{\partial\beta_{1}}\left[ \log\varphi_{ij}\left( \beta_{1} \right) \right] \right]$$

$$\Rightarrow\frac{\partial}{\partial\beta_{1}}\left[ \Phi_{i}\left( \beta_{1} \right) \right]=\Phi_{i}\left( \beta_{1} \right)\left[ -\sum_{j=1}^{J_{i}} \frac{{{AM}_{j}}^{2}}{{{SD}_{j}}^{2}}\frac{1}{\varphi_{ij}\left( \beta_{1} \right)}\frac{{nt_{\mathrm{ij}}\mathrm{SD}}_{j}^{2}}{\mathrm{AM}_{j}} \right]=-\Phi_{i}\left( \beta_{1} \right)\left[ \sum_{j=1}^{J_{i}} \frac{nt_{\mathrm{ij}}\mathrm{AM}_{j}}{\varphi_{ij}\left( \beta_{1} \right)} \right]$$

which gives rise to

$$\frac{\partial}{\partial\beta_{1}}p_{i}\left( \boldsymbol{r, \lambda} \right)= \sum_{n=1}^{\infty} {n*(-1)}^{n+1}\exp\left( {-n\beta}_{0} \right)\Phi_{i}\left( \beta_{1} \right) \left( \sum_{j=1}^{J_{i}} \frac{{t_{\mathrm{ij}}\mathrm{AM}}_{j}}{\varphi_{ij}\left( {n,\beta}_{1} \right)} \right)$$

Moreover,

$$\frac{\partial^{2}}{\partial{\beta_{0}}^{2}}p_{i}\left( \boldsymbol{r, \lambda} \right)= \sum_{n=1}^{\infty} n^{2}*\left( -1 \right)^{n}\exp\left( {-n\beta}_{0} \right)\Phi_{i}\left( \beta_{1} \right)$$

$$\frac{\partial^{2}}{\partial\beta_{1}\partial\beta_{0}}p_{i}\left( \boldsymbol{r, \lambda} \right)= \sum_{n=1}^{\infty} n^{2}*\left( -1 \right)^{n}\exp\left( {-n\beta}_{0} \right)\Phi_{i}\left( \beta_{1} \right)\left( \sum_{j=1}^{J_{i}} \frac{{t_{\mathrm{ij}}\mathrm{AM}}_{j}}{\varphi_{ij}\left( \beta_{1} \right)} \right)$$

and

$$\frac{\partial^{2}}{\partial{\beta_{1}}^{2}}p_{i}\left( \boldsymbol{r, \lambda} \right)= \sum_{n=1}^{\infty} {n^{2}*(-1)}^{n} exp\left( {-n\beta}_{0} \right)\Phi_{i}\left( \beta_{1} \right)\left( \sum_{j=1}^{J_{i}} \left[ \frac{{t_{\mathrm{ij}}\mathrm{SD}}_{j}}{\varphi_{ij}\left( \beta_{1} \right)} \right]^{2}+\left[ \sum_{j=1}^{J_{i}} \frac{{t_{\mathrm{ij}}\mathrm{AM}}_{j}}{\varphi_{ij}\left( \beta_{1} \right)} \right]^{2} \right)$$

Therefore,

$$LL\left( {\beta_{0},\beta}_{1} \right):=\log L\left( {\beta_{0},\beta}_{1} \right)=\sum_{i=1}^{N} {LL}_{i}\left( {\beta_{0},\beta}_{1} \right)$$

For $k=0, 1$

$$\frac{\partial}{\partial\beta_{k}}LL\left( {\beta_{0},\beta}_{1} \right)=\sum_{i=1}^{N} \frac{\partial}{\partial\beta_{k}} {LL}_{i}\left( {\beta_{0},\beta}_{1} \right)$$

and

$$\frac{\partial}{\partial\beta_{k}} {LL}_{i}\left( {\beta_{0},\beta}_{1} \right)=\frac{\left( -1 \right)^{1-y_{i}} *\frac{\partial}{\partial\beta_{k}}p_{i}\left( \boldsymbol{r, \lambda} \right)}{1-y_{i}+\left( -1 \right)^{1-y_{i}} *p_{i}\left( \boldsymbol{r, \lambda} \right)}$$

Let $f_{i}=1-y_{i}+\left( -1 \right)^{1-y_{i}} *p_{i}\left( \boldsymbol{r, \lambda} \right).$ The gradient $\text{G}$of the $LL$, with respect to $\beta_{0},\beta_{1}$ is given by:

| $\mathbf{G}=\sum_{i=1}^{N} \frac{\left( -1 \right)^{1-y_{i}}}{f_{i}} \left[ \begin{matrix} \frac{\partial}{\partial\beta_{0}}p_{i}\left( \boldsymbol{r, \lambda} \right) \\ \frac{\partial}{\partial\beta_{1}}p_{i}\left( \boldsymbol{r, \lambda} \right) \end{matrix} \right]$ | (A.1) |
| --- | --- |

Or

$$\mathbf{G}=\sum_{i=1}^{N} \frac{\left( -1 \right)^{1-y_{i}}}{f_{i}}\sum_{n=1}^{\infty} n*{(-1)}^{n+1} exp\left( {-n\beta}_{0} \right)\Phi_{i}\left( \beta_{1} \right)\left[ \begin{matrix} 1 \\ \sum_{j=1}^{J_{i}} \frac{{t_{\mathrm{ij}}\mathrm{AM}}_{j}}{\varphi_{ij}\left( n,\beta_{1} \right)} \end{matrix} \right]$$

and the hessian $\text{H}$ of the $LL$, with respect to $\beta_{0},\beta_{1}$ is the symmetric matrix given by

| $\mathbf{H}=\sum_{i=1}^{N} \frac{\left( -1 \right)^{1-y_{i}}}{{f_{i}}^{2}} \left[ \begin{matrix} H_{11,i} & H_{12,i} \\ H_{21,i} & H_{22,i} \end{matrix} \right]$ | (A.2) |
| --- | --- |

where for $l,k =1, 2$

$$H_{lk,i}=f_{i}*\frac{\partial^{2}}{\partial\beta_{l}\partial\beta_{k}}p_{i}\left( \boldsymbol{r, \lambda} \right)-\left( -1 \right)^{1-y_{i}}\frac{\partial}{\partial\beta_{l}}p_{i}\left( \boldsymbol{r, \lambda} \right)\frac{\partial}{\partial\beta_{k}}p_{i}\left( \boldsymbol{r, \lambda} \right)$$

**Appendix III: Gradient and Hessian of the log-likelihood of the conditional logistic regression**

$$L_{C,A}\left( \beta_{1} \right)= \prod_{k=1}^{S} \frac{\prod_{j=1}^{J_{0:k}} \left( 1-\frac{{\beta_{1}t_{0j:k}\mathrm{SD}}_{j}^{2}}{\mathrm{AM}_{j}} \right)^{- \frac{\mathrm{AM}_{j}^{2}}{\mathrm{SD}_{j}^{2}}}}{\prod_{j=1}^{J_{0:k}} \left( 1-\frac{{\beta_{1}t_{0j:k}\mathrm{SD}}_{j}^{2}}{\mathrm{AM}_{j}} \right)^{- \frac{\mathrm{AM}_{j}^{2}}{\mathrm{SD}_{j}^{2}}}+\sum_{i=1}^{N_{k}} \prod_{j=1}^{J_{i:k}} \left( 1-\frac{{\beta_{1}t_{ij:k}\mathrm{SD}}_{j}^{2}}{\mathrm{AM}_{j}} \right)^{- \frac{\mathrm{AM}_{j}^{2}}{\mathrm{SD}_{j}^{2}}}}=\prod_{k=1}^{S} L_{k}(\beta_{1})$$

where

$$L_{k}\left( \beta_{1} \right)=\frac{\prod_{j=1}^{J_{0:k}} \left( 1-\frac{{\beta_{1}t_{0j:k}\mathrm{SD}}_{j}^{2}}{\mathrm{AM}_{j}} \right)^{- \frac{\mathrm{AM}_{j}^{2}}{\mathrm{SD}_{j}^{2}}}}{\prod_{j=1}^{J_{0:k}} \left( 1-\frac{{\beta_{1}t_{0j:k}\mathrm{SD}}_{j}^{2}}{\mathrm{AM}_{j}} \right)^{- \frac{\mathrm{AM}_{j}^{2}}{\mathrm{SD}_{j}^{2}}}+\sum_{i=1}^{N_{k}} \prod_{j=1}^{J_{i:k}} \left( 1-\frac{{\beta_{1}t_{ij:k}\mathrm{SD}}_{j}^{2}}{\mathrm{AM}_{j}} \right)^{- \frac{\mathrm{AM}_{j}^{2}}{\mathrm{SD}_{j}^{2}}}}$$

Now for any function $f\left( \beta\right)$, denote $\log f\left( \beta\right)=Lf(\beta)$ so that we can write

$${LL}_{C,A}\left( \beta_{1} \right)=\log L_{C,A}\left( \beta_{1} \right)=\sum_{k=1}^{S} {LL}_{k}\left( \beta_{1} \right)$$

Then

$${LL}_{k}\left( \beta_{1} \right)=\sum_{j=1}^{J_{0:k}} - \frac{\mathrm{AM}_{j}^{2}}{\mathrm{SD}_{j}^{2}} \log\left( 1-\frac{{\beta_{1}t_{0j:k}\mathrm{SD}}_{j}^{2}}{\mathrm{AM}_{j}} \right)-\log\left[ \begin{aligned} \prod_{j=1}^{J_{0:k}} \left( 1-\frac{{\beta_{1}t_{0j:k}\mathrm{SD}}_{j}^{2}}{\mathrm{AM}_{j}} \right)^{- \frac{\mathrm{AM}_{j}^{2}}{\mathrm{SD}_{j}^{2}}}+\sum_{i=1}^{N_{k}} \prod_{j=1}^{J_{i:k}} \left( 1-\frac{{\beta_{1}t_{ij:k}\mathrm{SD}}_{j}^{2}}{\mathrm{AM}_{j}} \right)^{- \frac{\mathrm{AM}_{j}^{2}}{\mathrm{SD}_{j}^{2}}} \\ \end{aligned} \right]$$

Denote

$$\xi_{ij:k}\left( \beta_{1} \right)=\left( 1-\frac{{\beta_{1}t_{ij:k}\mathrm{SD}}_{j}^{2}}{\mathrm{AM}_{j}} \right), i=0,1,\cdots, N_{k}, \theta_{ij:k}\left( \beta_{1} \right)={\xi_{ij:k}\left( \beta_{1} \right)}^{-\frac{{{AM}_{j}}^{2}}{{{SD}_{j}}^{2}}}, i=0,1,\cdots, N_{k}$$

$$\Theta_{i:k}\left( \beta_{1} \right)=\prod_{j=1}^{J_{i:k}} \theta_{ij:k}\left( \beta_{1} \right), i=0,1,\cdots, N_{k}$$

So,

$$L_{C,A}\left( \beta_{1} \right)=\prod_{k=1}^{S} \frac{\Theta_{0:k}\left( \beta_{1} \right)}{\Theta_{0:k}\left( \beta_{1} \right)+\sum_{i=1}^{N_{k}} \Theta_{i:k}\left( \beta_{1} \right)}$$

Notice that

$${\xi_{ij:k}}^{'}\left( \beta_{1} \right)=-\frac{{t_{ij:k}\mathrm{SD}}_{j}^{2}}{{AM}_{j}}, i=0,1,\cdots, N_{k}$$

Using this, we have,

$$\frac{d}{d\beta_{1}}\left[ \sum_{j=1}^{J_{i:k}} \log\left( \Theta_{i:k}\left( \beta_{1} \right) \right) \right]=\sum_{j=1}^{J_{i:k}} \frac{t_{ij:k}{AM}_{j}}{\xi_{ij:k}\left( \beta_{1} \right)}$$

which implies

$${LL}_{k}'\left( \beta_{1} \right)=\left[ \sum_{j=1}^{J_{0:k}} \frac{{t_{0j:k}\mathrm{AM}}_{j}}{1-\frac{{\beta_{1}t_{0j:k}\mathrm{SD}}_{j}^{2}}{\mathrm{AM}_{j}}} \right]-\frac{T_{1}}{S_{1}}$$

where

$$T_{1}=\frac{d}{d\beta_{1}}\left[ \Theta_{0:k}\left( \beta_{1} \right)+\sum_{i=1}^{N_{k}} \Theta_{i:k}\left( \beta_{1} \right) \right], S_{1}=\Theta_{0:k}\left( \beta_{1} \right)+\sum_{i=1}^{N_{k}} \Theta_{i:k}\left( \beta_{1} \right)$$

Recall that $f^{'}\left( x \right)=f\left( x \right)\left( \log f(x) \right)'$ and so

$$\frac{d}{d\beta} \prod_{j=1}^{J} g_{j}\left( \beta\right)=\prod_{j=1}^{J} g_{j}\left( \beta\right)*\sum_{j=1}^{J} \frac{g_{j}'\left( \beta\right)}{g_{j}\left( \beta\right)}$$

Thus,

$$T_{1}=\prod_{j=1}^{J_{0:k}} \theta_{0j:k}\left( \beta_{1} \right)\sum_{j=1}^{J_{0:k}} \frac{t_{0j:k}{AM}_{j}}{\xi_{0j:k}\left( \beta_{1} \right)}+\sum_{i=1}^{N_{k}} \prod_{j=1}^{J_{i:k}} \theta_{ij:k}\left( \beta_{1} \right)\sum_{j=1}^{J_{i:k}} \frac{t_{ij:k}{AM}_{j}}{\xi_{ij:k}\left( \beta_{1} \right)}$$

Denote

$$\sum_{j=1}^{J_{i:k}} \frac{t_{ij:k}{AM}_{j}}{\xi_{ij:k}\left( \beta_{1} \right)}=\Lambda_{i:k}\left( \beta_{1} \right), i=0,1,\cdots N_{k}$$

so that

$$T_{1}=\Theta_{0:k}\left( \beta_{1} \right)\Lambda_{0:k}\left( \beta_{1} \right)+\sum_{i=1}^{N_{k}} \Theta_{i:k}\left( \beta_{1} \right)\Lambda_{i:k}\left( \beta_{1} \right)$$

Thus,

$${LL}_{C,A}'\left( \beta_{1} \right)=\sum_{k=1}^{S} {LL}_{k}'\left( \beta_{1} \right) =\Lambda_{0:k}\left( \beta_{1} \right)-\frac{\sum_{i=0}^{N_{k}} \Theta_{i:k}\left( \beta_{1} \right)\Lambda_{i:k}\left( \beta_{1} \right)}{\sum_{i=0}^{N_{k}} \Lambda_{i:k}\left( \beta_{1} \right)}$$

Now, denote

$$\frac{d}{d\beta_{1}}\left[ \Lambda_{i:k}\left( \beta_{1} \right) \right]=\left[ \sum_{j=1}^{J_{0:k}} \left( \frac{{t_{ij:k}\mathrm{SD}}_{j}}{1-\frac{{\beta_{1}t_{ij:k}\mathrm{SD}}_{j}^{2}}{\mathrm{AM}_{j}}} \right)^{2} \right]=\Psi_{i:k}(\beta_{1}), i=0, 1, 2,\cdots, N_{k}$$

Also,

$${LL}_{C,A}''\left( \beta_{1} \right)=\sum_{k=1}^{S} {LL}_{k}''\left( \beta_{1} \right)$$

where

$${LL''}_{k}\left( \beta_{1} \right)=\Psi_{0:k}(\beta_{1})-\frac{T_{2}*S_{1}-{T_{1}}^{2}}{{S_{1}}^{2}}$$

and

$$T_{2}=\frac{d}{d\beta_{1}}\left[ T_{1} \right]=\frac{d}{d\beta_{1}}\left[ \sum_{i=0}^{N_{k}} \Theta_{i:k}\left( \beta_{1} \right)\Lambda_{i:k}\left( \beta_{1} \right) \right]=\sum_{i=0}^{N_{k}} \Theta_{i:k}\left( \beta_{1} \right)\left[ \Psi_{i:k}\left( \beta_{1} \right)+{\Lambda_{i:k}}^{2}\left( \beta_{1} \right) \right]$$
